# Does an Old Art Suffice for New Problems?

**DOI:** 10.1007/s10699-015-9454-7

**Published:** 2015-10-17

**Authors:** Tsjalling Swierstra

**Affiliations:** Maastricht, Netherlands

**Keywords:** Ethical Technology Assessment, STS, Ethics, Art of life, Technological culture

## Abstract

In this review I argue that Puech draws on two important currents in modern thought: the criticism of the ontological and social priority of conflict, and the rehabilitation of praxis vis-à-vis theoria. Still, his plea for a non-confrontational art of living leaves important questions unanswered. What is the problem exactly? What does exactly count as (non)confrontational? What is non-confrontation exactly meant to solve? What is the antiposition here? And: how does this new (or rather: old) art of living relate to the political and ethical varieties of Technology Assessment?

Michel Puech’s plea for an old art of living in a new technological world is original and thought provoking. Not only does he manage to pull some of my personal strings—I happen to have an activist’s past in non-violent resistance—but more importantly: he manages to combine two important but separate currents in modern thought—one about ethics and politics, one about technology—into a new and original plea for a non-confrontational art of living with technology.

The first intellectual current has to do with the disparate but ubiquitous dismantling of (Herbert) Spencerian and neo-classical understandings of economics, society, politics, morality, and rationality. Whereas nineteenth and twentieth centuries’ theories prioritized struggle, conflict, and the competitive maximizing of individual utility, nowadays a growing body of work in economy, politics, biology, and ethics stresses that markets, societies, species, and individuals only flourish on the basis of non-confrontational capacities, dispositions, and relations, like trust, altruism, empathy, and care. A telltale sign of this paradigm shift is that whereas interpretations of Darwin previously stressed the role of competition, nowadays his theories on the role of sympathy and altruism draw the most attention. A good example here is provided by Frans de Waal’s work on primates as moral agents.

The second intellectual current is the so-called ‘practice turn’ in philosophy (Nicolini 2012). At least since Plato, the dominant philosophical schools were predicated on the assumption that the primary relation between humans and the world was one of contemplation and cognition. Practical engagement and interaction with the transient world was considered to be secondary, imperfect, and ‘lower class’. Now, I think a fair way to summarize modern philosophy in the last 150 years, is as a prolonged—albeit stagnating, partial, and hesitant—attempt to invert this Platonist theoria-praxis hierarchy. The results are as manifold as the philosophers whose names provide the milestones on this arduous path: Marx, Dewey, Heidegger, Wittgenstein, Merleau-Ponty, Searle, Foucault, Habermas, Rorty, Latour, and many many others. One defining hallmark of this practice turn is the attempt to weed out the asymmetric dichotomies in which one pole rules over the other. Such asymmetries are everywhere replaced by *symmetric relations* between two entities that are allowed to mutually shape each other: subject and object are for example now approached as the outcome of a mutually shaping relation. In the philosophy of technology we see exactly that movement. Classical philosophy of technology was deeply Platonist, and as a result deeply technophobic: its main problematic was how to avoid that objects would rise in revolt and end up ruling over subjects. By contrast, modern philosophy of technology takes its point of departure in the practical, embodied engagement of humans with the world. Part of that engagement is acknowledging and accepting that the material world shapes us as much as we shape it. Or more specifically: humans and technology, users and tools, constitute one another. The recent attention for ‘user creativity’ is part and parcel of this broader trend.

I see both currents at work in the background of Puech’s argument. On the one hand he moves away from struggle and conflict as the core paradigm for guiding/understanding social behaviour; on the other hand he accepts that technologies pervade our life world where they are shaping their users as much as being in turn shaped by them. There is no conflict between humans and technologies, as more Platonist philosophers of technology always assume. Combining the two currents allows him to invite us to forge new relations with technology. These new relations go beyond the ‘subject rules object/object rules subject’ problematic of classic philosophy of technology, pleading instead for a non-confrontational way of living with technology: not succumbing to or fighting against it, but embracing the inevitable mutual shaping of humans and artifacts through a lived strategy of selecting, adapting to, and reappropriating artifacts. Whereas fighting is propelled by negative reasons, the art of living is guided by positive reasons, e.g. sustainability, or user friendliness.

Although I share many of Puech’s inspirational sources and motivations, I admit to not being convinced—nor by the way he identifies the problem, nor by his solution, nor by the way he connects both. My first question is: what is the problem exactly? My second question is conceptual: what does exactly count as (non)confrontational? My third question is what non-confrontation is exactly meant to solve: what is the antiposition here? My fourth question, finally, is how this new (or rather: old) art of living relates to the previous Technology Assessment (TA) solutions, both political and ethical.

So, let’s first look at the problem that Puech’s argument is meant to solve. According to him the problem is that the modern life world is disruptive of the self, leaving it indecisive and lost in the midst of technologically created abundance and facilitation. Is this problem statement convincing? I have both factual and normative doubts. Factually: does this diagnosis hold for everyone? For whom is this disruption a problem, and how large is it really? I am pretty confident that not all social and economic strata are equally affected by the problems of abundance and facilitation. The modern self may well turn out to be predominantly middle or upper class. There is also a normative doubt: why should “disruption” be a problem exactly? Isn’t disruption also a precondition for learning and growth—as Puech himself acknowledges when he claims that contesting confrontation is okay because it is ethically disruptive? So, disruption in itself cannot be bad, except when equanimity is one’s life goal.

Then my second question: I am not convinced by the way Puech conceptualizes ‘confrontation’. What is to count as confrontation, and what is to count as non-confrontation? And how does confrontation relate to disruption of the self? For Puech, confrontation seems to equal opposing something for negative reasons, which I take to mean: for avoiding harms. But why is it less confrontation if one opposes something for positive reasons? And can one indeed in practice draw the distinction between negative and positive reasons so sharply? And is it indeed the case that we are disrupted by confrontation? For instance: if I travel, I do that to disrupt my daily routines, but not by seeking confrontation. In other instances I seek confrontation—for example by debating rather than dialoguing—exactly to avoid my convictions from being disrupted. Puech’s text is ambivalent here: one seems to be disrupted both by accepting technologies (as the ‘modern self’ does) *and* by critically rejecting them (as technology critics supposedly do). Maybe so, but then we need separate arguments for both groups. There is not a simple relation between confrontation and disruption of the self.

Thirdly, what problem is the strategy of non-confrontation meant to solve? What is the anti-position here? Presumably, confrontation to technology is the problem to be solved. But here the reader is left confused because the problem-diagnosis was not in terms of an over-dosis of confrontation, but rather in terms of an over-acceptance of technology. So, who is Puech fighting here: the witless consumers of technology who subsequently suffer from a loss of direction, or technology’s critics who mobilize negative reasons to confront technology? Is Puech’s argument directed against those who embrace the technology and then are disrupted by its abundance and facilitation? Or against those who oppose this technology? Or against both? To me it seems that the argument starts by diagnosing those who embrace technology—with all the disruptive consequences to the modern self—and then proceeds to offer a cure—non-confrontation to technology—that is offered for those who don’t embrace, but oppose, technology.

Finally, how does the proposed art of living relate to the “previous two periods of TA”: political critique and ethical assessment? Is “existential awareness” meant to *complement* the first two, or to *replace* them? In the first option, it is unclear how the non-confrontational ethos can be compatible with more confrontational ways to relate to technology as ethical assessment and political critique. I think Puech leans towards the second option in which individual existential strategies *replace* ethical and political ones, because he appropriates the slogan ‘Don’t confront the system, but bypass it’. Exactly at this point my concerns with ancient Asian wisdom surface. Admittedly, I am in no position to assess the wisdom of ages and of other cultures. But my ignorance doesn’t stop me from having a well-known concern: isn’t Buddhism too adaptive, too conformist, too dis-sociated from our mundane concerns? Isn’t Buddhism an art of living designed for a world that is mostly beyond our control and—therefore—beyond our responsibility? And that thus seeks redemption through individual strategies of accommodating to the inevitable? But—and that is the central paradox of modernity—technology has made life more malleable in the same degree that it has presented itself as inevitable. Technology has opened up the world to our interventions in the same move that it has presented itself as inaccessible for external interventions. So, I am not convinced that a pre-technological art of living will provide us with solutions to the problems of a technological culture.

I am well aware that we are living in a world so complex as to defy naïve enlightenment hopes. We have lived through too many scientific and technological horrors to still attach a naïve belief to the grand narratives of modernity. Be that as it may, we also live in a world of grand scale problems, that need grand scale solutions. In other words: we cannot afford to substitute politics and ethics by individual strategies of existential awareness. For instance: we won’t solve climate change by growing our own vegetables—even though it is tempting to reduce our energies to such human-scale activities that provide instant gratification.

So, although Puech and I seem to share intellectual sources, we don’t agree about the problem or about its solution. For one, I think that confrontation is not bad per se, and that disruption is to be valued more than in the traditional arts of living, as it is also a source of learning and creativity. I don’t think there is a direct line between confrontation and negative disruption. Rather, I envisage a world that is safe enough to allow a lot of disruption, which indeed would mean that negative confrontation is replaced by positive, productive confrontation. And I would like to see a world in which mundane tinkering with technologies would directly inform the ethical assessment thereof, as well as the political decisions about how to provide structural conditions for good technology. In such a way, we could do both justice to the way people select, adapt, and re-appropriate the technologies that help shape their life world, and to the ways people find to shape their communal fate.
